# Consequences of Oviposition Site Choice for Geckos in Changing Environments

**DOI:** 10.3390/biology11091281

**Published:** 2022-08-29

**Authors:** Theja Abayarathna, Jonathan K. Webb

**Affiliations:** 1 Department of Biological Sciences, Faculty of Applied Sciences, Rajarata University of Sri Lanka, Mihintale 50300, Sri Lanka; 2 School of Life Sciences, University of Technology Sydney, Broadway, NSW 2007, Australia

**Keywords:** heatwave, lizard, climate change, phenotypic plasticity, maternal nest site choice

## Abstract

**Simple Summary:**

Most lizards lay eggs inside nests where developing embryos experience large temperature fluctuations. As females do not incubate eggs, the embryos can experience lethally high temperatures during heatwaves. Thus, future changes in the frequency and intensity of summer heatwaves may threaten lizard populations. However, variation in female nest site choice might buffer the embryos in some nests from high temperatures. In this study, we incubated eggs of the velvet gecko under two fluctuating temperature regimes to mimic the temperatures experienced inside currently used sun-exposed (“warm”: mean = 25.4 °C; range = 16.5–35.5 °C) and shaded (“cold”: mean = 23.3 °C; 17.5–30.5 °C) communal nest sites. We found that warm-incubated hatchlings hatched 15 days earlier, on average, and were smaller than their cold-incubated clutch mates. We released the hatchlings to the wild, and monitored their survival over six months. Egg incubation treatment did not influence the survival of hatchlings. This result is reassuring, because even if air temperatures increase by 2 °C in future, some currently used shaded nests will provide thermal regimes that are suitable for embryonic development. Variation in female nest site choice may therefore allow some populations of velvet geckos to persist in changing environments.

**Abstract:**

Most lizards lay eggs inside nests where embryos experience daily fluctuations in temperature. As embryos are sensitive to exposure to high temperatures, increases in nest temperatures may pose a risk to lizards. In the velvet gecko *Amalosia lesueurii*, nest temperatures are positively correlated with air temperatures, so nests may get hotter in future. However, maternal variation in oviposition site choice might buffer populations from future warming. To evaluate the consequences of oviposition site choice, we incubated eggs under two fluctuating temperature regimes that mimicked temperatures experienced inside sun-exposed (“warm”: mean = 25.4 °C; range = 16.5–35.5 °C) and shaded (“cold”: mean = 23.3 °C; 17.5–30.5 °C) communal nests. We measured the phenotypic traits of hatchlings, released them to the wild, and monitored their survival over 6 months. Warm-incubated hatchlings hatched 15 days earlier, on average, and were smaller than their cold-incubated clutch mates. Incubation treatment did not influence the apparent survival of hatchlings. Hence, even if air temperatures increase by 2 °C in future, thermal regimes inside some currently used shaded nests will be suitable for embryo development. Maternal variation in nest site choice may therefore allow southern populations of the velvet gecko to persist in changing environments.

## 1. Introduction

A female’s decision about where and when to oviposit can influence egg hatching success, the quality and sex of her offspring, and can also affect a female’s lifetime reproductive success. Maternal oviposition site choices can thereby influence demographic processes that can affect the longer-term persistence of populations [1,2]. In reptiles, key evolutionary drivers of oviposition site choice include maximizing maternal survival, maximizing egg survival, enhancing offspring traits that influence juvenile survival, and providing suitable habitats for hatchlings [3]. Although a key assumption of life history theory is that females should choose oviposition sites that are optimal for offspring development [4], females may choose suboptimal sites to maximize their own survival at the expense of offspring quality. For example, during the nesting season, females of the freshwater turtle *Emydura macquarii* are vulnerable to predation by introduced red foxes. An experimental study showed that females adjusted their choice of egg laying sites depending on the level of predation risk. At sites where foxes were common, females laid their nests closer to the water, where eggs were more likely to be eaten by predators, or drown during floods. By contrast, at sites where foxes were removed, females laid their nests further from the water, at sites where nests were less likely to be destroyed by predators or impacted by floods [5]. Thus, maternal nest site choice may not always maximize offspring fitness.

Determining the consequences of maternal oviposition site choice is crucial for understanding how future environmental changes may affect the persistence of populations. Reptiles are threatened by anthropogenic changes such as habitat loss, fragmentation, and overharvesting [6,7,8], and because they are ectothermic, they are also at risk from climate warming [9]. Embryonic life stages are particularly sensitive to increasing temperatures because most reptiles lack parental care, and abandon eggs after oviposition. During the incubation period, eggs within natural nests can experience marked fluctuations in temperature [10,11,12,13]. Therefore, a mothers’ choice of nest site will dictate the thermal (and hydric) conditions experienced by her embryos [14]. Incubation temperatures can affect embryo survival [15] and hatchling traits such as size, shape, sex, cold tolerance, and behavior [10,16,17,18,19,20,21]. Due to land use changes such as urbanization and forest clearing, increases in ambient temperatures wrought by the urban heat island effect may lead to increases in nest temperatures in many lizard species [13]. More broadly, climate modelers have predicted that summer heatwaves will increase in intensity and duration in future [22]. For lizard species with temperature dependent sex-determination (TSD), in which nest temperatures influence the sex of the offspring, warming temperatures pose a risk of skewed sex ratios, which could lead to population declines [23]. Therefore, it is important to consider how natural variation in nest sites influences offspring phenotypes and survival [24,25]. While we know much about incubation-induced phenotypic variation, we know less about how that variation influences hatchling survival in the wild [26,27].

Here, we investigate the consequences of nest site selection for velvet geckos *Amalosia lesueurii.* Velvet geckos are vulnerable to high temperatures because females lay eggs in communal nests in rock crevices, and nest temperatures are positively correlated with air temperatures [28]. Thus, if the intensity and duration of summer heatwaves increases in the future, as predicted by climate modelers [22,29], then nest temperatures may shift upwards. Although velvet geckos are not reported to have TSD, they are nonetheless at risk from warming because incubation-induced changes in hatchling survival rates can increase the risk of local extinctions [28]. Previous demographic analyses have assumed that females lack plasticity in nesting behavior [28]; however, at small spatial scales (<1 ha) there is considerable variation in the physical characteristics (nest depth, aspect, rock thickness, and canopy cover) and thermal profiles of communal nests [30,31]. This spatial variation in nest site thermal regimes may provide nesting females with suitable nest sites in future, potentially buffering them from climatic variation.

To investigate the effects of thermal variation in nest site temperatures, we incubated eggs of the velvet gecko *Amalosia lesueurii* under fluctuating thermal regimes to mimic temperature profiles experienced inside currently used shaded (“cold”) and sun-exposed (“warm”) nest sites. After the eggs hatched, we measured the length and mass of the hatchlings in the laboratory. We then individually marked the hatchlings, released them at field sites, and carried out a mark-recapture study to assess whether incubation environments or phenotypic traits influenced the apparent survival of hatchlings in the wild.

## 2. Materials and Methods

### 2.1. Study Species

Lesueur’s Velvet Gecko (*Amalosia lesueurii*) inhabits sandstone and granite rock outcrops in eastern Australia and occurs from southeastern New South Wales to southeastern Queensland [29]. In southern populations, adults mature at age 2 to 3 years, and live for up to 13 years in the wild [32]. Females attain larger snout-vent lengths than males (means of 64.1 versus 57.7 mm), and sex ratios of adults were female biased with twice as many females as males being captured over a three year period [32]. Hatchlings cannot be accurately sexed, but juvenile and adult males possess a single row of rugose scales on either side of the tail base which is lacking in females. Females lay their eggs inside communal nests in rock crevices, and nesting crevices have different physical and thermal attributes than non-used potential nest sites [27,30,31]. Females oviposit two eggs per clutch in spring from late October to early November with an incubation period of 80–120 days [32]. Temperatures recorded inside nine communal nests from Morton National Park in 2006–2007 ranged from 9.5–44.5 °C (mean = 22.7 °C) during the incubation period [25].

### 2.2. Site Descriptions and Collection of Adult Females

We carried out a mark-recapture study of velvet geckos at two sites, one near Nowra, NSW, 170 km south of Sydney, and another in Dharawal National Park, 60 km south of Sydney, NSW. Both sites contained sun-exposed sandstone rock outcrops surrounded by dry sclerophyll forest. Both sites were restored in 2009 with 50 identical artificial rocks (512 mm long × 352 mm wide × 46 mm thick) constructed from fiber-reinforced cement that were placed in sun-exposed locations. These rocks provide thermal regimes and crevices that are very similar to those found under natural rocks [33], and were rapidly colonized by velvet geckos [34]. We chose these habitat restoration sites because they support large populations of velvet geckos, and have communal egg laying sites that are used by females. The artificial rocks also provide identical shelter sites for geckos, thereby reducing variation in shelter site characteristics, which could potentially influence survival of geckos.

In October 2015, we collected gravid females near communal nests at each study site by carefully turning all artificial and natural rocks. When we captured a gecko, we recorded the rock’s unique number (that was painted on its underside) and its location (with a GPS) so that we could return the females to their exact site of capture. Females were transported to the University of Technology Sydney and were housed individually in clear ventilated containers (Sistema, Auckland, New Zealand, 220 × 150 × 60 mm) with an identical shelter (plastic half pipe), and moist vermiculite as an oviposition site. Cages were placed in a constant temperature room (22 °C) with 12:12 light cycle. One end of each cage was placed on timer-controlled heating racks to create a daytime thermal gradient of 22–32 °C. Geckos had access to water ad libitum and were fed crickets twice weekly. Females were held until they had laid eggs and were then released at the exact site of capture.

### 2.3. Egg Incubation Experiment

After oviposition eggs were weighed (to 0.01 g) and placed in 100 mL autoclaved glass jars containing autoclaved moist vermiculite, which we sealed with cling wrap. One egg from each clutch of two eggs was randomly selected and allocated to one of the two incubation treatments. Eggs were incubated inside two programmable temperature incubators (Panasonic MIR-154-PE, Panasonic Healthcare Co., Gunma Japan, with 10 step functions) programmed to mimic fluctuating temperatures experienced inside current sun-exposed “warm” (mean = 25.4 °C; range = 16.5–35.5 °C) and shaded “cold” (mean = 23.3 °C; range = 17.5–30.5 °C) nest sites (Figure S1, Supplementary Materials). Incubation treatment temperatures were based on field data collected from 9 communal nests in 2006–2007; these nests have been used as communal egg laying sites by female velvet geckos since 1992 (Webb, personal observation).

After eggs hatched, we weighed each hatchling (to 0.01 g), and measured their snout-vent length (SVL) and tail length (TL) with a ruler (to nearest mm, see Table S1, Supplementary Materials). Each hatchling was housed in a ventilated plastic cage (220 × 155 × 61 mm) with a paper substrate, water dish and a plastic shelter (PVC half pipe, 100 × 55 × 25 mm). Cages were placed on timer-controlled heating racks to provide a thermal gradient during the daytime (22–32 °C) dropping to room temperature at night. Geckos were fed small crickets twice weekly and were released at the sites where their mothers were captured after experiments were completed.

### 2.4. Release and Mark-Recapture

Prior to release, we gave each hatchling a unique toe-clip to allow subsequent identification. Previous studies indicate that toe-clipping causes minimal stress to lizards [35] and does not appear to affect the longer-term survival of hatchling velvet geckos [34,36]. Each gecko was released at the study site where its mother was captured; the rationale for this was that had the females not been transported to the lab, they would have laid eggs in the communal nests on the study site. At each site, each hatchling was placed underneath a vacant artificial rock to reduce variation in habitat structure and temperature that could potentially affect hatchling survival. Prior to release, we measured the temperatures of the rock substrate and the underside of each rock with an infrared thermometer (Cool Tech CT-663, spot diameter = 13 mm) to ensure that the rock temperatures were within the thermal tolerance limits of geckos. At our field site in Nowra, we released 37 warm-incubated hatchlings on 23 March 2016 and 34 cold-incubated hatchlings on 6 April 2016. At the field site in Dharawal, we released 14 warm incubated and 21 cold incubated hatchlings on the 6 April 2016. To estimate hatchling survival, we visited each study site between April 2016 to September 2016 and carefully turned all artificial rocks and natural rocks that could be safely lifted without causing a back injury. We focused on estimating survival during the first six months of life because during this period hatchlings are sedentary, and shelter under one or two rocks [33], so that estimates of survival are likely to reflect true survival rather than emigration. Sampling dates for Nowra were 21/04/2016, 11/05/2016, 8/06/2016, 29/06/2016, 15/07/2016 and 20/10/2016, while sampling dates for Dharawal were 14/04/2016, 12/05/2016, 18/05/2016, 15/06/2016, 19/07/2016, 26/07/2016 and 8/09/2016. For each captured gecko, we recorded the rock number, and measured the gecko’s SVL and TL, to the nearest mm (with a ruler), and recorded the toe-clip.

### 2.5. Statistical Analyses

To investigate whether incubation temperature affected the morphology of hatchlings, we used two factor ANOVAs, with location and treatment as factors, and SVL, TL and mass as the dependent variables. To test whether incubation treatment or body size influenced gecko survival, we ran Cormack-Jolly Seber (CJS) models in Program MARK v 9.0 [37]. Data for each study site (Nowra and Dharawal) were analyzed separately. For each site, we ran two analyses. The first analysis tested whether incubation treatment affected survival, and the second analysis tested whether incubation temperature and/or body size influenced survival. This step wise analysis was necessary because we first had to check whether the assumptions of mark-recapture were met, and MARK is unable to do this when covariates are present in the input file. For the first analysis, we included incubation treatment (cold versus warm) as a group factor in the input file, and ran several models to see whether survival and recapture rates were constant, group-dependent, or time-dependent. To verify that the CJS assumptions were met, we tested the goodness of fit of the most parameterized model in our candidate model set using a bootstrap GOF test. For both data sets, the bootstrap GOF test showed that model assumptions were met (Dharawal, *p* = 0.86; Nowra, *p* = 0.80), so c-hat was not adjusted.

For the second analysis, we included the covariates SVL, TL and mass in the input files (Tables S2 and S3, Supplementary Materials) so that we could test whether survival was influenced by incubation treatment, snout-vent length, tail length, or mass. We then ran a series of models in MARK to test the following a priori hypotheses: (1) survival is dependent on incubation treatment; (2) there is directional selection on body size (the “bigger is better” hypothesis) (3) there is time varying directional selection on body size. We also ran equivalent survival models, in which recapture probability was constant, group dependent, time dependent, or was influenced by one of the covariates. We included these models to explore the possibility that incubation treatment or body size might influence recapture rates. Candidate models were ranked based on their AIC values and associated AIC weights [34]. In general, models with delta AIC < 2.0 are considered to be supported by the data, while models with delta AIC > 4 have little support.

## 3. Results

### 3.1. Hatching Success and Incubation Period

Hatching success did not differ between the two incubation treatments. In the warm incubation treatment, 54 eggs of 78 eggs hatched successfully, while 56 of 79 eggs hatched from the cold incubation treatment (χ^2^ = 0.05, *p* = 0.82, Table 1). Warm incubated eggs hatched from the 25 January to the 18 February 2016, while the cold incubated eggs hatched from the 2 February to the 4 March 2016. On average, warm-incubated eggs hatched 15 days earlier (mean incubation period 86.24 ± 8.67 days) than cold incubated eggs (mean incubation period 101.83 ± 8.25 days, F _1, 108_ = 92.52, *p* < 0.001).

### 3.2. Hatchling Morphology

Hatchlings from the cold incubation treatment were larger than hatchlings from the warm incubation treatment (Table 1). Cold incubated hatchlings had larger snout-vent lengths (incubation treatment F_1, 106_ = 9.82, *p* = 0.002; location F_1, 106_ = 1.87, *p* = 0.17; interaction F_1, 106_ = 3.22, *p* = 0.08), longer tails (incubation treatment F_1, 103_ = 5.10, *p* = 0.03; location (F_1, 103_ = 0.07, *p* = 0.80, interaction: F_1, 103_ = 0.34, *p* = 0.56), and were heavier (F_1, 106_ = 4.11, *p* = 0.045; location F_1, 106_ = 0.14, *p* = 0.71, interaction F_1, 106_ = 0.04, *p* = 0.85) than warm-incubated hatchlings.

### 3.3. Effects of Incubation Temperature on Hatchling Survival

**Figure 1:**
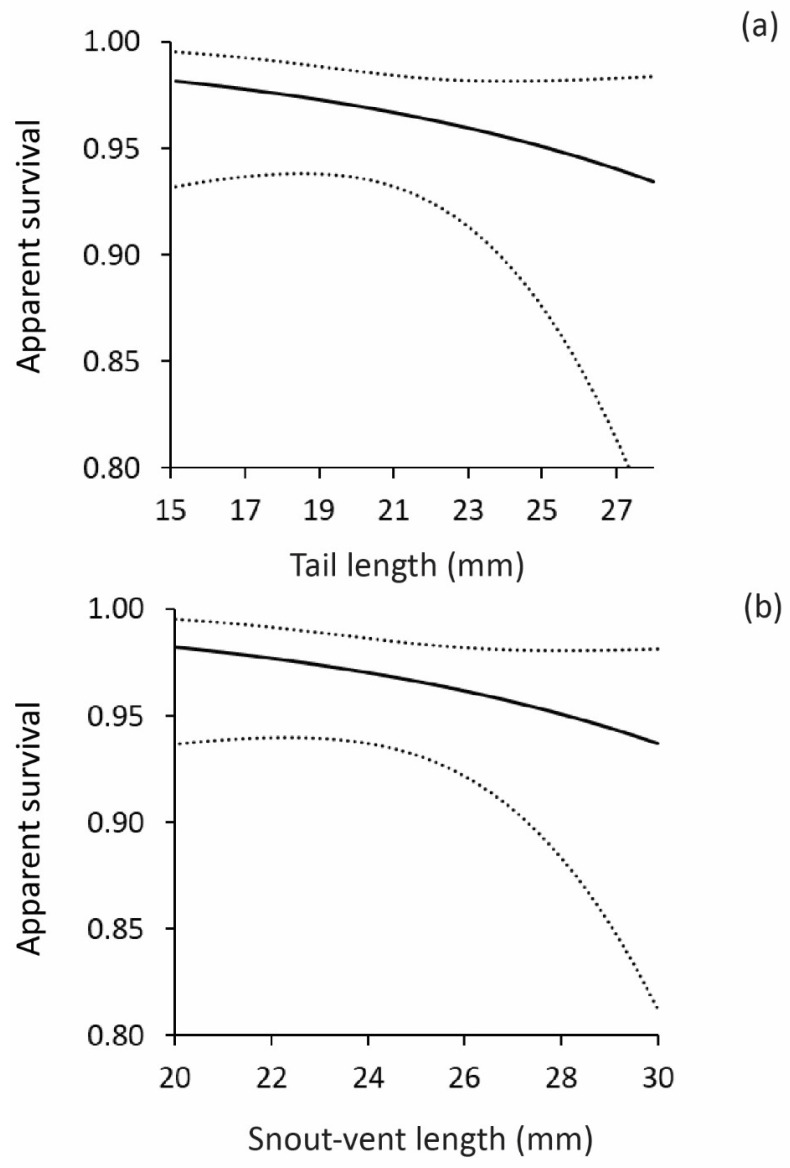
Relationship between apparent survival of hatchling geckos and: (**a**) tail length and (**b**) snout-vent length. Dotted lines show 95% CIs.

For the Dharawal data set, the results of the CJS survival analyses showed that incubation temperature did not affect lizard survival. The best supported model was one in which survival was influenced by time and tail length, and recapture was constant. From this model, recapture rates were 0.55 (SE = 0.045), but survival rates varied over time, and were lowest between the period between release and the first sampling occasion (s = 0.97, SE = 0.012), and higher in the other time periods (estimates ranged from 0.98 to 1.0). Plots of survival rates versus TL showed that hatchlings with longer tails had lower rates of survival during the first time period (Figure 1a). An identical pattern was observed for SVL, with larger lizards exhibiting lower survival rates than smaller lizards (Figure 1b) during the period between release and the first sampling occasion. None of the other models were well supported by the data (delta AICc > 2; Table 2).

For the Nowra data set, there was no evidence that incubation treatment influenced hatchling survival (Table 3). The best-supported model was one in which survival rates varied with time and body mass, and recapture rates were group dependent. In this model, recapture rates were higher for warm-incubated lizards (mean = 0.56, SE = 0.09) than cold-incubated lizards (mean = 0.28, SE = 0.08). Interestingly, body mass influenced survival rates, but only during the period between release and resampling, where heavier lizards had lower survival than lighter lizards. Another two models also received equivalent support (delta AICc < 2, Table 3). The second model had time-dependent survival rates and group-dependent recapture rates, while the third model was equivalent to model one except that recapture rates were constant. None of the other models were well supported (Table 3).

## 4. Discussion

Future changes in climate pose challenges for lizards. To understand how such changes may affect velvet geckos, we incubated eggs under thermal regimes that mimicked nest temperatures inside currently used shaded nests (cold treatment) and sun-exposed nests (warm treatment). We found that incubation temperature influenced the incubation period, and body size of hatchlings, but did not affect egg hatching success, or apparent survival of hatchlings.

Egg hatching success was similar between treatments, but warm incubated eggs hatched 16 days earlier than the cold incubated eggs. This finding agrees with previous experimental studies; in general, incubation under temperatures typically experienced inside natural nests does not affect hatchling success, but higher temperature incubation results in shorter incubation periods [26,27]. The timing of oviposition and hatching can influence the survival of juvenile lizards, but there are no clear patterns [35]. In cold climates, earlier hatching may allow hatchlings to grow and store fat, which may in turn influence overwinter survival [36,37]. In other species, later hatching may be advantageous [38]. In our study species, hatchling geckos settle under small rocks near communal nests [33]. In hot years, hatching early may be a hindrance for geckos if they emerge from nests during summer heatwaves, when rock temperatures are lethally high [28]. During heatwaves, hatchlings would be forced to use cooler crevices, which might compromise their growth [39], or make them more vulnerable to predators [40]. By contrast, hatchlings from colder nests sites may avoid this thermally stressful time period [28].

We found that cold-incubated hatchlings were larger than their warm incubated clutch mates. This result agrees with previous studies on velvet geckos, which found that hatchlings from eggs incubated at high temperatures were smaller and lighter than hatchlings from eggs incubated at colder temperatures [20,21,28]. In lizards, hatchlings from higher temperature incubation tend to be smaller than hatchlings from colder incubation temperatures [26,27]. The physiological mechanism responsible for this size difference appears to be linked to incubation period and yolk conversion. In most reptiles, lower incubation temperatures generate longer incubation periods that lead to increased conversion of yolk to tissue, which results in larger hatchlings [41].

Although incubation temperatures influenced hatchling body size, there was no evidence that incubation temperature affected the apparent survival of hatchling geckos at the field sites during the six-month study (Table 2 and Table 3). As hatchlings take up to three years to reach maturity [32], longer term studies are necessary to evaluate whether incubation temperature affects survival to adulthood. Nonetheless, there was strong evidence that hatchling body size influenced apparent survival, at least during some time intervals, but selection on body size was not in the direction predicted by the ‘bigger is better’ hypothesis which predicts that larger hatchlings should have higher survival than smaller conspecifics [42]. In lizards, larger individuals may have higher survival because they are able to capture larger prey, establish territories in better habitats, outcompete smaller lizards, or better withstand food shortages via stored lipids in the tail [43,44,45]. For example, in side-blotched lizards (*Uta stansburiana*), larger juveniles had a survival advantage, and occupied better quality territories, than smaller conspecifics [43]. In our study, we found evidence for temporal selection on body size, with larger lizards having lower survival than smaller lizards during the period between release to the field and the first sampling trip. This finding could reflect size-related differences in survival, as has been reported previously for lizards [46], or size-related differences in the dispersal of hatchlings. For example, if larger lizards moved further than smaller lizards, they may have moved away from the study sites, and thus, were never recaptured. Although we cannot distinguish between these two possibilities, studies on common lizards found that body condition influenced the propensity of juveniles to disperse, with individuals in good condition more likely to disperse than individuals in poor condition [47].

## 5. Conclusions

We now return to the question of how velvet geckos may cope with future changes in climate. In our experiment, we incubated eggs under thermal regimes that mimicked nest temperatures inside currently used shaded nests (cold treatment) and sun-exposed nests (warm treatment). Despite the 2.1 °C difference between the mean temperatures in our experimental treatments, we found no evidence that incubation temperatures influenced egg hatching success or offspring survival. These results are reassuring, because even if air temperatures increase by 2 to 3 °C in future, as predicted by climate modelers [48], temperatures within shaded nest sites would remain suitable for offspring development, even during summer heatwaves [31]. Shaded nests, with temperatures similar to those used in our experiments, accounted for 30% of nests used by females from a southern population (Morton National Park) during the summer of 2019–2020. Although this was one of the hottest summers on record, maximum temperatures in shaded nests did not exceed 33.7 °C [31]. Thus, provided that there is maternal variation in nest-site choice, and sufficient shaded nest sites, southern velvet gecko populations should be buffered against future change. By contrast, more northerly velvet gecko populations may be at risk. For example, in one northern population, 50% of nests that were monitored during 2019–2020 experienced maximum temperatures that exceeded the species CT_max_ [31]. Clearly, more research is needed to evaluate the effects of thermal spikes on egg viability and hatchling phenotypes [49]. Finally, our research has conservation implications for the endangered broad-headed snake (*Hoplocephalus bungaroides)*. Juveniles of this species feed mostly on velvet geckos [50], so understanding how velvet gecko populations will cope with future environmental change will be crucial for managing and conserving populations of both predators and prey.

## Figures and Tables

**Table 1:** The effect of cold versus warm incubation treatment on egg hatching success, incubation period, and hatchling snout-vent length, tail length and wet body mass. Standard deviations of means are shown in parentheses.

	Cold Incubation	Warm Incubation
	(*n* = 56)	(*n* = 54)
Hatching success (%)	70.9	70.1
Incubation period (d)	101.83 (8.25)	86.24 (8.67)
Snout-vent length (mm)	27.05 (1.94)	25.78 (1.69)
Tail length (mm)	23.06 (3.01)	21.25 (4.25)
Wet body mass (g)	0.48 (0.06)	0.46 (0.06)

**Table 2:** Results of survival analyses used to compare rates of apparent survival (s) and recapture (p) for warm-incubated and cold-incubated hatchlings from Dharawal. The candidate models were ranked based on their AICc values and associated AICc weights; models with delta AICc < 2.0 have the greatest statistical support. The table also shows the model likelihood, number of parameters (N), and model deviance. SVL and TL indicate the covariates snout-vent length and tail length, respectively.

Model	AICc	Delta AICc	AICc Weights	Likelihood	N	Deviance
s (time + TL) p (constant)	283.2314	0	0.51811	1	5	272.6489
s (time + SVL) p (constant)	285.2356	2.0042	0.1902	0.3671	6	272.4121
s (time + mass) p (constant)	286.38	3.1486	0.10733	0.2072	6	273.5565
s (constant) p (time)	288.074	4.8426	0.04601	0.0888	8	270.634
s (constant) p (constant)	289.0449	5.8135	0.02832	0.0547	2	284.9317
s (SVL) p (constant)	289.0882	5.8568	0.02771	0.0535	3	282.8596
s (TL) p (constant)	289.6272	6.3958	0.02116	0.0408	3	283.3986
s (incubation) p (time)	290.3492	7.1178	0.01475	0.0285	9	270.531

**Table 3:** Results of survival analyses used to compare rates of apparent survival (s) and recapture (p) for warm-incubated and cold-incubated hatchling geckos that were captured at Nowra in 2016. The candidate models were ranked based on their AICc values and associated AICc weights; models with delta AICc < 2.0 have the greatest statistical support. The table also shows the model likelihood, number of parameters (N), and model deviance. Incubation refers to incubation treatment (warm vs. cold); SVL and TL indicate the covariates snout-vent length and tail length, respectively.

Model	AICc	Delta AICc	AICc Weights	Likelihood	N	Deviance
s (time + mass) p (incubation)	226.2744	0	0.39835	1	7	211.1431
s (time) p (incubation)	227.9026	1.6282	0.17648	0.443	6	215.0626
s (time + mass) p (constant)	228.0635	1.7891	0.16284	0.4088	5	217.4695
s (time + SVL) p (incubation)	228.3592	2.0848	0.14046	0.3526	7	213.2279
s (time + SVL) p (constant)	230.0278	3.7534	0.06098	0.1531	5	219.4337
s (time) p (constant	231.179	4.9046	0.0343	0.0861	5	220.5849
s (time + TL) p (incubation)	233.5323	7.2579	0.01057	0.0265	7	218.401
s (time + TL) p (constant)	234.5871	8.3127	0.00624	0.0157	5	223.993

## Data Availability

Data supporting reported results can be found in the Supplementary Materials.

## Supplementary Materials

The following supporting information can be downloaded at: https://www.mdpi.com/article/10.3390/biology11091281/s1. Figure S1, temperature profiles of the cold and warm egg incubation treatments. Table S1, raw data for egg incubation experiment; Table S2, raw data for field mark-recapture sampling at Dharawal National Park; Table S3, raw data for field mark-recapture sampling at Nowra.
