# Supplementary material for: Consequences of Oviposition Site Choice for Geckos in Changing Environments
Source: Biology (Basel). 2022 Aug 29;11(9):1281. doi: 10.3390/biology11091281 (PMC9495809; doi:10.3390/biology11091281)
Supplement: Supplementary file 1 [file biology-11-01281-s001.zip › biology-1860872-supplementary.pdf]

Supplementary Information

Egg incubation treatments

Females laid clutches of two eggs in the lab. Shortly after eggs were laid, we placed eggs singly inside 100 mL glass jars filled with moist vermiculite (water potential of 200 KPa). We sealed each jar with plastic food wrap to prevent the eggs from desiccating and randomly allocated one egg from each clutch produced by each female to each of two programmable incubators (Panasonic MIR 154, 10 step functions). We programmed incubators to mimic fluctuating temperatures experienced during summer inside current sun-exposed "warm" (mean = 25.4 °C; range = 16.5–35.5 °C) and shaded "cold" (mean = 23.3 °C; range = 17.5–30.5 °C) nest sites. Incubators had glass doors, and lights in the room were programmed to come on at sunrise, and go off at sunset each day. Temperatures were programmed to cycle on a daily basis, but minima and maxima increased as summer progressed, and there were hotter periods to simulate the temperatures that can occur during heatwaves (Figure S1). We recorded the temperature inside each incubator by placing four miniature data loggers (Thermochron DS1922L-F5#, accuracy of ± 0.5 °C) inside100 ml glass jars which were filled with egg incubation media and sealed with cling wrap. The glass jars containing the data loggers were positioned at the front and rear of the top and bottom shelves of each incubator. Due to an electrical fault, the cold incubator stopped functioning for 3 days, so temperatures drifted to ambient at that time until the fault was fixed (Figure S1a).

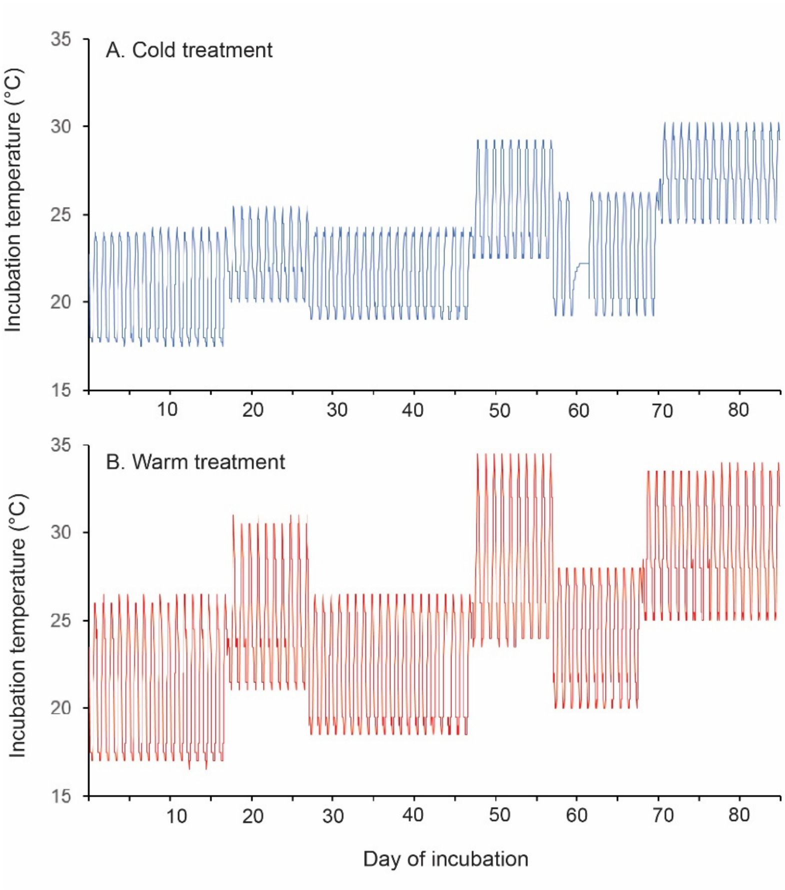

Figure S1. Temperature profiles of the cold (a) and warm (b) incubation treatments.

Table S1. Morphology of hatchlings from the egg incubation experiment. The term 'DT' indicates that a hatchling dropped its tail, and so no tail length was recorded for that individual.

Location	Incubation treatment	Snout-vent length (mm)	Tail length (mm)	Mass (g)
Dharawal	Cold	25	22	0.48
Dharawal	Cold	27	19	0.47
Dharawal	Cold	28	22	0.41
Dharawal	Cold	26	24	0.48
Dharawal	Cold	26	22	0.43
Dharawal	Cold	24	21	0.31
Dharawal	Cold	30	23	0.52
Dharawal	Cold	29	23	0.56
Dharawal	Cold	27	25	0.67
Dharawal	Cold	26	22	0.42
Dharawal	Cold	28	23	0.46
Dharawal	Cold	26	28	0.52
Dharawal	Cold	26	DT	0.48
Dharawal	Cold	28	23	0.42
Dharawal	Cold	23	19	0.46
Dharawal	Cold	27	23	0.41
Dharawal	Cold	22	21	0.50
Dharawal	Cold	26	27	0.54
Dharawal	Cold	25	23	0.49
Dharawal	Cold	29	23	0.47
Dharawal	Cold	26	24	0.50
Dharawal	Cold	26	19	0.49
Dharawal	Warm	26	10	0.46
Dharawal	Warm	26	22	0.46
Dharawal	Warm	25	22	0.48
Dharawal	Warm	25	24	0.50
Dharawal	Warm	26	20	0.41
Dharawal	Warm	26	20	0.46
Dharawal	Warm	30	26	0.54
Dharawal	Warm	27	28	0.50
Dharawal	Warm	26	22	0.39
Dharawal	Warm	27	24	0.61
Dharawal	Warm	27	26	0.46
Dharawal	Warm	28	24	0.47
Dharawal	Warm	25	24	0.48
Dharawal	Warm	27	23	0.48
Dharawal	Warm	22	20	0.33
Dharawal	Warm	22	17	0.36
Nowra	Cold	27	20	0.44
Nowra	Cold	28	25	0.54
Nowra	Cold	30	24	0.44

Nowra	Cold	27	24	0.50
Nowra	Cold	29	27	0.56
Nowra	Cold	25	22	0.56
Nowra	Cold	28	24	0.55
Nowra	Cold	25	23	0.5
Nowra	Cold	28	16	0.52
Nowra	Cold	26	DT	0.39
Nowra	Cold	29	25	0.4
Nowra	Cold	29	26	0.44
Nowra	Cold	27	21	0.47
Nowra	Cold	28	25	0.37
Nowra	Cold	29	25	0.51
Nowra	Cold	27	24	0.46
Nowra	Cold	25	26	0.48
Nowra	Cold	29	28	0.53
Nowra	Cold	26	10	0.51
Nowra	Cold	28	22	0.52
Nowra	Cold	29	25	0.48
Nowra	Cold	26	24	0.49
Nowra	Cold	30	23	0.51
Nowra	Cold	28	23	0.50
Nowra	Cold	24	23	0.42
Nowra	Cold	26	23	0.58
Nowra	Cold	31	24	0.40
Nowra	Cold	29	26	0.41
Nowra	Cold	26	24	0.52
Nowra	Cold	27	21	0.54
Nowra	Cold	28	24	0.59
Nowra	Cold	26	22	0.41
Nowra	Cold	31	29	0.52
Nowra	Cold	24	21	0.39
Nowra	Warm	28	21	0.48
Nowra	Warm	28	22	0.54
Nowra	Warm	25	12	0.46
Nowra	Warm	26	21	0.56
Nowra	Warm	24	9	0.45
Nowra	Warm	25	21	0.58
Nowra	Warm	25	20	0.43
Nowra	Warm	26	23	0.47
Nowra	Warm	26	21	0.49
Nowra	Warm	26	25	0.51
Nowra	Warm	26	22	0.49
Nowra	Warm	28	25	0.45
Nowra	Warm	25	20	0.39
Nowra	Warm	22	16	0.37
Nowra	Warm	24	20	0.43
Nowra	Warm	23	10	0.41

Nowra	Warm	28	23	0.42
Nowra	Warm	24	DT	0.41
Nowra	Warm	24	22	0.43
Nowra	Warm	24	20	0.48
Nowra	Warm	26	20	0.44
Nowra	Warm	23	21	0.38
Nowra	Warm	26	22	0.39
Nowra	Warm	25	27	0.46
Nowra	Warm	27	26	0.52
Nowra	Warm	26	24	0.48
Nowra	Warm	26	21	0.43
Nowra	Warm	26	21	0.46
Nowra	Warm	27	23	0.44
Nowra	Warm	26	21	0.49
Nowra	Warm	25	20	0.39
Nowra	Warm	27	25	0.51
Nowra	Warm	27	21	0.46
Nowra	Warm	28	25	0.50
Nowra	Warm	29	28	0.51
Nowra	Warm	25	22	0.36
Nowra	Warm	26	22	0.42

Table S2. Mark recapture data for geckos released at a site in Dharawal National Park. Each row indicates the mark-recapture data for an individual gecko. The first eight characters code for the mark-recapture data, with '1' indicating that the individual was captured, and '0' indicating that the individual was not captured. After the space, the next two characters designate the incubation treatment, with '1 0' coding for the cold incubation treatment and '0 1'coding for the warm incubation treatment. The covariates for snout-vent length, tail length, and mass appear after the coding for incubation treatment.

10010000 1 0 24 19 31;
11111111 1 0 25 23 55;
11101110 1 0 25 22 49;
10101101 1 0 25 22 48;
10000000 1 0 23 21 48;
10111110 1 0 22 20 27;
10100000 1 0 26 20 49;
11111000 1 0 30 25 47;
10000000 1 0 26 21 50;
11010000 1 0 23 19 42;
11110011 1 0 24 23 49;
11011110 1 0 25 21 40;
11011111 1 0 25 22 39;

10000000 1 0 21 19 19;
10000000 1 0 27 24 46;
10110000 1 0 23 22 39;
10100000 1 0 30 28 47;
10010000 1 0 25 19 50;
10000001 1 0 21 15 48;
10001000 1 0 22 21 36;
10000100 1 0 23 20 48;
10100000 0 1 25 23 49;
10010000 0 1 24 21 57;
11011110 0 1 24 17 42;
10000000 0 1 24 18 68;
10000000 0 1 23 19 45;
10111111 0 1 22 15 45;
10000000 0 1 27 21 44;
11111111 0 1 26 19 58;
10111101 0 1 22 21 30;
10000001 0 1 20 16 32;
10001111 0 1 27 22 57;
10000000 0 1 25 21 52;
10000000 0 1 27 22 54;
11000100 0 1 26 19 56;

Table S3. Mark recapture data for geckos released at a site near Nowra. Each row indicates the mark-recapture data for an individual gecko. The first eight characters code for the mark-recapture data, with '1' indicating that the individual was captured, and '0' indicating that the individual was not captured. After the space, the next two characters designate the incubation treatment, with '0 1' coding for the cold incubation treatment and '1 0'coding for the warm incubation treatment. The covariates for snout-vent length, tail length, and mass appear after the coding for incubation treatment.

1000000 0 1 27 24 48;
1000000 0 1 26 21 50;
1000000 0 1 29 25 49;
1000100 0 1 25 19 45;
1001110 0 1 25 19 39;
1001010 0 1 24 22 50;
1000000 0 1 25 23 47;
1000000 0 1 25 20 48;
1000000 0 1 25 22 45;
1001000 0 1 27 25 48;
1000000 0 1 26 22 49;
1000000 0 1 25 23 43;
1000000 0 1 25 22 39;

1000000 0 1 27 24 56;
1000000 0 1 27 22 49;
1000000 0 1 25 18 41;
1000000 0 1 26 23 52;
1000000 0 1 24 21 52;
1000000 0 1 27 23 48;
1000000 0 1 23 15 40;
1000000 0 1 24 18 44;
1000000 0 1 25 23 62;
1001111 0 1 25 19 45;
1100000 0 1 27 23 34;
1000000 0 1 23 17 34;
1100000 0 1 25 16 38;
1000000 0 1 23 16 34;
1000010 0 1 27 22 33;
1000000 0 1 23 21 48;
1010000 0 1 24 19 49;
1000000 0 1 24 23 46;
1000000 0 1 25 21 47;
1000000 0 1 25 22 50;
1000000 0 1 28 21 46;
1110010 1 0 22 22 45;
1110001 1 0 24 9 45;
1110000 1 0 26 21 46;
1111111 1 0 24 25 41;
1000000 1 0 27 21 51;
1100000 1 0 22 9 36;
1000000 1 0 26 22 50;
1000000 1 0 25 23 40;
1000000 1 0 28 21 47;
1000000 1 0 24 18 33;
1000000 1 0 27 23 53;
1000000 1 0 24 22 52;
1000000 1 0 25 21 49;
1000000 1 0 25 19 57;
1000000 1 0 23 22 58;
1110001 1 0 24 22 32;
1001000 1 0 22 19 42;
1000000 1 0 23 9 41;
1000000 1 0 26 21 45;
1000000 1 0 29 24 46;
1000000 1 0 28 24 60;
1000000 1 0 24 19 46;
1000000 1 0 25 19 42;
1010111 1 0 27 23 51;

1000000 1 0 26 22 52;
1110001 1 0 24 19 48;
1000000 1 0 22 19 47;
1000000 1 0 26 24 51;
1000000 1 0 24 19 41;
1000000 1 0 26 23 39;
1000000 1 0 25 21 58;
1000000 1 0 24 20 48;
1000000 1 0 25 16 30;
1000000 1 0 26 21 49;
1000000 1 0 27 16 43;
1000000 1 0 24 21 39;
1100000 1 0 23 19 46;
